# Supplementary material for: Observing anyonization of bosons in a quantum gas
Source: Nature. 2025 May 28;642(8066):53–7. doi: 10.1038/s41586-025-09016-9 (PMC12137132; doi:10.1038/s41586-025-09016-9)
Supplement: Supplementary file 1 — Supplementary Information [file 41586_2025_9016_MOESM1_ESM.pdf]

nature portfolio

https://doi.org/10.1038/s41586-025-09016-9

Supplementary information

Observing anyonization of bosons in a quantum gas

In the format provided by the authors and unedited

Supplementary Materials of "Observing anyonization of bosons in a quantum gas"

Sudipta Dhar,[1,*] Botao Wang,[2,3,*] Milena Horvath,[1,*] Amit Vashisht,[2,3] Yi Zeng,[1] Mikhail B. Zvonarev,[4] Nathan Goldman,[2,3,5] Yanliang Guo,[1,†] Manuele Landini,[1,‡] and Hanns-Christoph Nägerl[1,§]

[1] *Institut für Experimentalphysik und Zentrum für Quantenphysik, Universität Innsbruck, Technikerstraße 25, Innsbruck, 6020, Austria*
[2] *Center for Nonlinear Phenomena and Complex Systems, Université Libre de Bruxelles, CP 231, Campus Plaine, B-1050 Brussels, Belgium*
[3] *International Solvay Institutes, 1050 Brussels, Belgium*
[4] *Université Paris-Saclay, CNRS, LPTMS, 91405 Orsay, France*
[5] *Laboratoire Kastler Brossel, Collège de France, CNRS, ENS-Université PSL, Sorbonne Université, 11 Place Marcelin Berthelot, 75005 Paris, France*

S1. EXACT SOLUTION VIA BETHE ANSATZ

Here, we consider the problem of an impurity interacting with a one-dimensional TG gas via a short-ranged δ-function potential of arbitrary strength $g_{\uparrow\downarrow}$. The TG gas can be mapped to a gas of spin-polarized free fermions. The system is governed by the Hamiltonian[1]

$$\hat{H} = \frac{1}{2m}\sum_{i=1}^{N}\hat{P}_i^2 + \frac{1}{2m}\hat{P}_\downarrow^2 + g_{\uparrow\downarrow}\sum_{i=1}^{N}\delta(x_i - x_\downarrow), \tag{S1}$$

where x_i and $\hat{P}_i$ are the position and momentum of the i-th background particle, respectively. The position and momentum of the impurity are denoted by $x_\downarrow$ and $\hat{P}_\downarrow$, respectively. All particles are assumed to have the same mass m. The dimensionless LL interaction strength is given by $\gamma_{\uparrow\downarrow} = \frac{mg_{\uparrow\downarrow}}{\hbar^2\rho}$, where $\rho = \frac{N}{L}$ is the density of the background gas and L is the system-size. The Fermi momentum, defined as $k_{\mathrm{F}} = \pi\rho$, is directly proportional to the 1D density ρ. This model (S1) is integrable for any value of $g_{\uparrow\downarrow}$ and can be solved via Bethe ansatz[2]. Some calculations are easier in the mobile impurity reference frame. This frame is accessed via the Lee-Low-Pines (LLP) transformation[3], sometimes called the polaron transformation, which is frequently used in polaron physics. For simplicity, we set $g_{\uparrow\downarrow} \equiv g$ and $\hbar = m = 1$ in the following.

A. Lee-Low-Pines transformation

The key object is the operator

$$\mathcal{Q} = e^{i\hat{P}_\uparrow \hat{x}_\downarrow}. \tag{S2}$$

Here, $x_\downarrow$ is the position of the impurity and $P_\uparrow$ is the total momentum of the host particles. The transformation of an arbitrary operator $\mathcal{O}$ from the laboratory to the mobile impurity reference frame is given by

$$\mathcal{O} \to \mathcal{O}_\mathcal{Q} = \mathcal{Q}\mathcal{O}\mathcal{Q}^{-1}. \tag{S3}$$

The LLP transformation does not affect the momentum of the host particles but changes the momentum operator of the impurity

$$\hat{P}_{\uparrow\mathcal{Q}} = \hat{P}_\uparrow, \qquad \hat{P}_{\downarrow\mathcal{Q}} = \hat{P}_\downarrow - \hat{P}_\uparrow. \tag{S4}$$

---

* These authors contributed equally to this work.
† yanliang.guo@uibk.ac.at
‡ manuele.landini@uibk.ac.at
§ christoph.naegerl@uibk.ac.at

Therefore, the total momentum of the system in the mobile impurity reference frame reads

$$\hat{P}_{\mathcal{Q}} = \hat{P}_{\downarrow}. \tag{S5}$$

Let us apply $\mathcal{Q}$ to the wavefunction. Recall that

$$e^{a\frac{d}{dx}} f(x) = f(x+a). \tag{S6}$$

As a result,

$$\mathcal{Q}\Psi_Q(x_\downarrow, x_1, \dots, x_N) = \Psi_Q(x_\downarrow, x_1 + x_\downarrow, \dots, x_N + x_\downarrow) = e^{iQx_\downarrow}\Psi_Q(0, x_1, \dots, x_N). \tag{S7}$$

Here Ψ_Q is the wavefunction in the original frame, the subscript Q indicates the value of the total momentum of the system. Note that we work with fermions in the continuum, hence our system is translationally invariant. The shift of all coordinates is achieved by the action of the momentum operator as follows from Eq. (S6). This is how we got the right hand side of Eq. (S7). We can rewrite Eq. (S7) as

$$\Psi_Q(x_\downarrow, x_1, \dots, x_N) = e^{iQx_\downarrow}\Psi_Q(0, x_1 - x_\downarrow, \dots, x_N - x_\downarrow) \equiv e^{iQx_\downarrow} f_Q(y_1, \dots, y_N), \tag{S8}$$

where $y_j = x_j - x_\downarrow$, $j = 1, \dots, N$.

The function $f_Q(y_1, \dots, y_N)$ is the wavefunction of the system in the mobile impurity reference frame. Working with its first-quantized representation, we aim at doing an exact calculation for finite N. Hence, we impose periodic boundary conditions to take into account finite-size effects. For the following calculations, we consider the case where N is odd.

B. Bethe ansatz solution for arbitrary coupling

The Hamiltonian S1 in the mobile impurity frame transforms to,

$$\hat{H}_Q = \frac{1}{2}\sum_{i=1}^{N}\hat{P}_i^2 + \frac{1}{2}(\hat{P}_\downarrow - \hat{P}_\uparrow)^2 + g\sum_{i=1}^{N}\delta(y_i). \tag{S9}$$

Thus, any gas particle in the impurity frame is scattered by the impurity particle positioned at the origin. The wavefunctions of the problem in the impurity frame look particularly simple. They are just Slater determinants[4]

$$f_Q(y_1, \dots, y_N) = \frac{Y}{\sqrt{N!L^N}} \begin{vmatrix} e^{ik_1 y_1} & \dots & e^{ik_{N+1} y_1} \\ \vdots & \ddots & \vdots \\ e^{ik_1 y_N} & \dots & e^{ik_{N+1} y_N} \\ \nu(k_1) & \dots & \nu(k_{N+1}) \end{vmatrix}, \qquad 0 \le y_j \le L, \tag{S10}$$

where

$$\nu(q) = \frac{g}{2}\frac{1}{q - \frac{g}{2}(\Lambda + i)}. \tag{S11}$$

The factor Y ensures the normalization condition. The set of quasi-momenta $k_1, \dots, k_{N+1}$ satisfies a system of nonlinear equations (Bethe equations)

$$\cot\frac{k_j L}{2} = \frac{2k_j}{g} - \Lambda, \qquad j = 1, 2, \dots, N+1. \tag{S12}$$

Here Λ is a free parameter, whose value is fixed by requiring

$$Q = \sum_{j=1}^{N+1} k_j. \tag{S13}$$

That is, the sum of the quasi-momenta give the total momentum (which is an observable). In the system with finite number of particles the total momentum is quantized as usual,

$$Q = \frac{2\pi}{L}n, \qquad n = 0, \pm 1, \pm 2, \dots. \tag{S14}$$

The energy of the state is

$$E_{\mathrm{F}} = \frac{1}{2}\sum_{j=1}^{N+1} k_j^2. \tag{S15}$$

Let us recall how Eqs. (S12) are obtained. The function (S10) has to be continuous,

$$f_Q(y_1,\ldots,y_N)\Big|_{y_j=0}^{y_j=L} = 0. \tag{S16}$$

Its first derivative should experience a jump such that the second derivative generates the terms $g\delta(y_j)$:

$$-\partial_{y_j} f_Q(y_1,\ldots,y_N)\Big|_{y_j=0}^{y_j=L} = g f_Q(y_1,\ldots,y_j=0,\ldots,y_N). \tag{S17}$$

Substituting the function (S10) into these two equations we get the desired equations (S12).

The form (S10) is valid when all y_j are contained in the interval from zero to L. The expression, for example, for y_j in the interval from L to $2L$ is not given by Eq. (S10). We discuss how to extend Eq. (S10) from the interval $0 \le y_j \le L$ for the particular case $g \to \infty$ in the next section.

C. Bethe ansatz solution in the limit of infinite repulsion

The form of Eq. (S10) further simplifies in the limit of infinite repulsion, $g \to \infty$. There, the function (S11) becomes momentum-independent,

$$\nu(q) = -\frac{1}{\Lambda + i}, \qquad g \to \infty \tag{S18}$$

and the wavefunction (S10) takes the form

$$f_Q(y_1,\ldots,y_N) = \frac{\tilde{Y}}{\sqrt{N!L^N}} \begin{vmatrix} e^{ik_1y_1} & \ldots & e^{ik_{N+1}y_1} \\ \vdots & \ddots & \vdots \\ e^{ik_1y_N} & \ldots & e^{ik_{N+1}y_N} \\ 1 & \ldots & 1 \end{vmatrix}, \qquad g \to \infty \tag{S19}$$

in the domain $0 \le y_j \le L$. The Bethe equations (S12) also simplify a lot:

$$\cot\frac{k_jL}{2} = -\Lambda, \qquad j = 1,2,\ldots,N+1, \qquad g \to \infty. \tag{S20}$$

We see that the quasi-momenta k_j are quantized like free fermions plus a shift, same for all k_js from a given set:

$$k_j = q_j + \frac{\mu}{L}, \qquad j = 1,2,\ldots,N+1, \qquad g \to \infty, \tag{S21}$$

where $\mu = -2\tan(\Lambda)$ and q_j are free-fermion momenta

$$q_j = \frac{2\pi}{L} n_j, \qquad n_j = 0,\pm1,\pm2,\ldots. \tag{S22}$$

We therefore have

$$f_Q(y_1,\ldots,y_N) = \frac{\tilde{Y}}{\sqrt{N!L^N}} \prod_{j=1}^{N} e^{i\mu y_j/L} \begin{vmatrix} e^{iq_1y_1} & \ldots & e^{iq_{N+1}y_1} \\ \vdots & \ddots & \vdots \\ e^{iq_1y_N} & \ldots & e^{iq_{N+1}y_N} \\ 1 & \ldots & 1 \end{vmatrix}, \qquad g \to \infty \tag{S23}$$

in the domain $0 \le y_j \le L$.

It is worth mentioning that the function f_Q remains far from trivial even in the $g \to \infty$ limit, despite the seemingly "free-fermion" form of the expressions (S19) and (S23). This is because each plane wave, e^{ik_jy}, still does not satisfy periodic boundary conditions, that is, $e^{ik_jL} \neq 1$.

It is important to keep in mind that the function (S19) is defined in the domain $0 \le y_j \le L$ (which means that the gas particles are positioned to the right of the impurity). Let us now extend the definition to the case where some particles are placed to the left of the impurity, that is, we tackle the domain $-L \le y_j \le L$ by defining the function extension as $\tilde{f}_Q$. We require periodicity of $\tilde{f}_Q$ on a ring of circumference L:

$$\tilde{f}_Q(y_1, \dots, y_j - L, \dots, y_N) = \tilde{f}_Q(y_1, \dots, y_j, \dots, y_N), \quad j = 1, \dots, N. \tag{S24}$$

Having Eq. (S19) and using Eq. (S24) we propose

$$\tilde{f}_Q(y_1, \dots, y_N) = \frac{\tilde{Y} e^{-i\frac{\mu}{2}\prod_{j=1}^{N}\mathrm{sgn}(y_j)}}{\sqrt{N! L^N}} \begin{vmatrix} e^{ik_1 y_1} & \dots & e^{ik_{N+1} y_1} \\ \vdots & \ddots & \vdots \\ e^{ik_1 y_N} & \dots & e^{ik_{N+1} y_N} \\ 1 & \dots & 1 \end{vmatrix}, \qquad g \to \infty. \tag{S25}$$

It is the factor containing the sign functions that ensures the validity of Eq. (S25) in the whole domain $-L \le y_j \le L$. Coming back to the laboratory frame we get for this wavefunction

$$\Psi_Q(x_\downarrow, x_1, \dots, x_N) = \frac{\tilde{Y} e^{-i\frac{\mu}{2}\prod_{j=1}^{N}\mathrm{sgn}(x_j - x_\downarrow)}}{\sqrt{N! L^N}} \times \begin{vmatrix} e^{ik_1 x_1} & \dots & e^{ik_{N+1} x_1} \\ \vdots & \ddots & \vdots \\ e^{ik_1 x_N} & \dots & e^{ik_{N+1} x_N} \\ e^{ik_1 x_\downarrow} & \dots & e^{ik_{N+1} x_\downarrow} \end{vmatrix}, \qquad g \to \infty. \tag{S26}$$

valid in the domain $-L/2 \le x_j \le L/2$, $j = 1, \dots, N, \downarrow$. Recall that Eqs. (S25) and (S26) are connected by the transformation (S7). The function (S26) is antisymmetric with respect to any permutation of the host particles.

At this point, let us summarize our knowledge about the wavefunctions and about the spectrum in the $g \to \infty$ limit. Equation (S26) is a Slater determinant. This way, the impurity problem at infinite repulsion behaves just as the free fermion one. Indeed, if any two of the coordinates from the set $x_1, \dots, x_N, x_\downarrow$ take the same value, the determinant vanishes regardless of the values of the quasi-momenta $k_1, \dots, k_{N+1}$. This is how fermions should behave. The function (S26) is antisymmetric with respect to any permutation of the host particles. What is really amazing about Eq. (S26) is its periodicity, that is, Ψ takes the same values at $x_j = -L/2$ and $x_j = L/2$, $j = 1, \dots, N, \downarrow$ in the case of $k_1, \dots, k_{N+1}$ quantized according to the Bethe equations (S20) and (S14), despite the fact that each plane wave is not periodic in this interval, $e^{ik_j L} = e^{i\mu}$. We now reformulate the problem in the language of the second quantization.

D. Anyon-fermion mapping of the $g \to \infty$ problem and second quantization

The parameter μ is related to the total momentum $\hbar Q$ as $Q = k_\mathrm{F}(1 + \frac{\mu}{\pi})$. The wavefunction for each value of μ can be written in the second-quantized form as follows

$$|\Psi_Q\rangle = \frac{1}{\sqrt{N!}} \int_0^L dx_\downarrow dx_1 \cdots dx_N \Psi_Q(x_\downarrow, x_1, \dots, x_N) \psi_\mathrm{A}^\dagger(x_\downarrow) \psi^\dagger(x_1) \cdots \psi^\dagger(x_N)|0\rangle. \tag{S27}$$

Here, ψ is the fermion destruction operator and ψ_A behaves as an impenetrable anyon with respect to ψ,

$$\psi_\mathrm{A}(x_\downarrow)\psi(x) - e^{-i(\mu+\pi)\mathrm{sgn}(x_\downarrow - x)}\psi(x)\psi_\mathrm{A}(x_\downarrow) = 0, \tag{S28}$$

while the host particles behave with respect to each other as free fermions. The impurity-host exchange phase is zero for Q=0 and π for $Q = k_\mathrm{F}$ as anticipated in the main text. Note that the momentum distribution of the impurity $n_\downarrow(k)$ is the same whether the host particles are free fermions or a TG gas and $n_\downarrow(k)$ can be expressed through a correlation function of 1D impenetrable anyons[5].

S2. ANYON-HUBBARD MODEL IN A NUTSHELL

In this section, we briefly review the properties of the above-mentioned anyon-Hubbard model, which is a paradigmatic model to describe anyons in 1D lattices[6,7],

$$\hat{H}_\mathrm{AHM} = -J \sum_\ell \left(\hat{a}_\ell^\dagger \hat{a}_{\ell+1} + h.c. \right) + \frac{U}{2} \sum_\ell \hat{n}_\ell \left(\hat{n}_\ell - 1 \right), \tag{S29}$$

where J and U denote the tunneling amplitude and the on-site interaction between anyons, respectively, and $\hat{n}_\ell = \hat{a}_\ell^\dagger \hat{a}_\ell$ is the number operator at site ℓ. The *anyonic* operators $\hat{a}_\ell$ obey the generalized commutation relations

$$\hat{a}_j^\dagger \hat{a}_k - e^{-i\theta \mathrm{sgn}(j-k)} \hat{a}_k^\dagger \hat{a}_j = \delta_{jk}, \quad \hat{a}_j \hat{a}_k - e^{-i\theta \mathrm{sgn}(j-k)} \hat{a}_k \hat{a}_j = 0. \tag{S30}$$

The above relations can be obtained by means of a fractional version of the Jordan-Wigner transformation, i.e., via the anyon-boson mapping

$$\hat{a}_\ell = \hat{b}_\ell e^{i\theta N_\ell}, \quad \hat{N}_\ell = \sum_{j=1}^{\ell-1} \hat{n}_j, \tag{S31}$$

where $\hat{b}_\ell$ are bosonic operators and obey the bosonic commutation relation $[\hat{b}_j, \hat{b}_k^\dagger] = \delta_{jk}, [\hat{b}_j, \hat{b}_k] = 0 = [\hat{b}_j^\dagger, \hat{b}_k^\dagger]$. Note that the above transformation gives the same number operators, i.e., $\hat{n}_\ell = \hat{a}_\ell^\dagger \hat{a}_\ell = \hat{b}_\ell^\dagger \hat{b}_\ell$.

Combining the anyon-boson mapping (S31) with Eq. (S29), the anyon-Hubbard Hamiltonian can be expressed in terms of bosonic operators as

$$\hat{H}_{\mathrm{AHM}}^{\mathrm{B}} = -J \sum_\ell \left(\hat{b}_\ell^\dagger \hat{b}_{\ell+1} e^{i\theta \hat{n}_\ell} + h.c. \right) + \frac{U}{2} \sum_\ell \hat{n}_\ell \left(\hat{n}_\ell - 1 \right). \tag{S32}$$

Here, we will be interested in the quasi-momentum distribution. One has to distinguish the quasi-momentum distribution in terms of bosonic operators from that using anyonic operators,

$$\langle \hat{n}_k^{\mathrm{b}} \rangle = \frac{1}{L} \sum_{\ell\ell'} e^{ik(\ell-\ell')} \langle \hat{b}_\ell^\dagger \hat{b}_{\ell'} \rangle, \tag{S33}$$

$$\langle \hat{n}_k^{\mathrm{a}} \rangle = \frac{1}{L} \sum_{\ell\ell'} e^{ik(\ell-\ell')} \langle \hat{a}_\ell^\dagger \hat{a}_{\ell'} \rangle. \tag{S34}$$

In the hardcore limit, the bosonic Hamiltonian (S32) will be independent of the statistical angle θ, i.e.,

$$\hat{H}_{\mathrm{AHM}}^{\mathrm{B}} \overset{U\to\infty}{\longrightarrow} \hat{H}_{\mathrm{AHM}}^{\mathrm{B}} = -J \sum_\ell \left(\hat{b}_\ell^\dagger \hat{b}_{\ell+1} + h.c. \right). \tag{S35}$$

Thus, computing the quasi momentum of bosons $\langle \hat{n}_k^{\mathrm{b}} \rangle$ would give a θ-independent quasi-momentum distribution[8].

However, the anyonic nature can be revealed by computing the anyonic quasi-momentum distribution $\langle \hat{n}_k^{\mathrm{a}} \rangle$, because the anyonic correlation $\langle \hat{a}_\ell^\dagger \hat{a}_{\ell'} \rangle$ are modified by the Jordan-Wigner transformation. To clarify this, we can explicitly write the anyonic correlation as

$$\langle \hat{a}_\ell^\dagger \hat{a}_{\ell'} \rangle = \langle \hat{b}_\ell^\dagger e^{i\theta(\hat{N}_{\ell'} - \hat{N}_\ell)} \hat{b}_{\ell'} \rangle = \begin{cases} \langle \hat{b}_\ell^\dagger \hat{b}_{\ell'} e^{i\theta(\hat{N}_{\ell'} - \hat{N}_\ell)} \rangle, & \ell' \geq \ell \\ \langle \hat{b}_\ell^\dagger \hat{b}_{\ell'} e^{i\theta(\hat{N}_{\ell'} - \hat{N}_\ell + 1)} \rangle, & \ell' < \ell. \end{cases} \tag{S36}$$

Here, $\langle \bullet \rangle \equiv \langle \Psi | \bullet | \Psi \rangle$ represents the expectation value with respect to the ground state $|\Psi\rangle$ of the Hamiltonian (S32), which can be expressed in the Fock state basis $\{|\psi_i\rangle\}$ as

$$|\Psi\rangle = \sum_j c_j |\psi_j\rangle, \quad |\psi_j\rangle = |n_1^j, n_2^j, \cdots, n_L^j\rangle \tag{S37}$$

with complex coefficients c_i, $n_\ell^i = \{0, 1\}$ in the hard-core limit, and L being the total site number of the chain. In this case, the above anyonic correlation (S36) can be rewritten as

$$\langle \hat{a}_\ell^\dagger \hat{a}_{\ell'} \rangle = \begin{cases} \sum_{i,j} c_i^* c_j \langle \psi_i | \hat{b}_\ell^\dagger \hat{b}_{\ell'} | \psi_j \rangle e^{i\theta(N_{\ell'}^j - N_\ell^j)}, & \ell' \geq \ell, \\ \sum_{i,j} c_i^* c_j \langle \psi_i | \hat{b}_\ell^\dagger \hat{b}_{\ell'} | \psi_j \rangle e^{-i\theta(N_\ell^j - N_{\ell'}^j - 1)}, & \ell' < \ell. \end{cases} \tag{S38}$$

with $N_\ell^j = \sum_j$. Here, the (N-dependent) phase factors in Eq. (S36) and (S38) are attributed to the contribution from the Jordan-Wigner transformation, which transfers the bosonic correlation $\langle \hat{b}_\ell^\dagger \hat{b}_{\ell'} \rangle$ to be the anyonic one $\langle \hat{a}_\ell^\dagger \hat{a}_{\ell'} \rangle$.

To benchmark the anyonic correlations from the AHM with the experiment, we consider the experimental impurity momentum distribution at $\theta/\pi = 0.53(2)$ as an example. As shown in Fig. S1, the agreement between the experimental data and the anyonic momentum distribution obtained from the AHM improves for large system sizes and low filling.

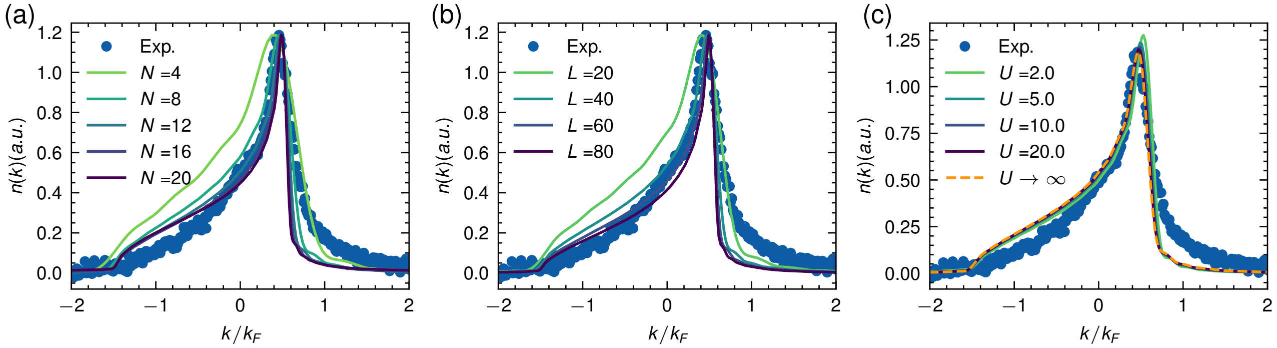

Figure S1. **Benchmarking the experimental data by the AHM.** (a) Effect of total host particle number at $L = 40$ and $U \to \infty$. (b) Effect of the system size at filling 1/4 at $U \to \infty$. (c) Effect of the on-site interaction U at $L = 40, N = 10$. The blue dots are the experimental data for $\theta/\pi = 0.53(2)$; the solid lines are the prediction from the AHM. Note that in (a,b), the peak has been rescaled to match the amplitude of the experimental data.

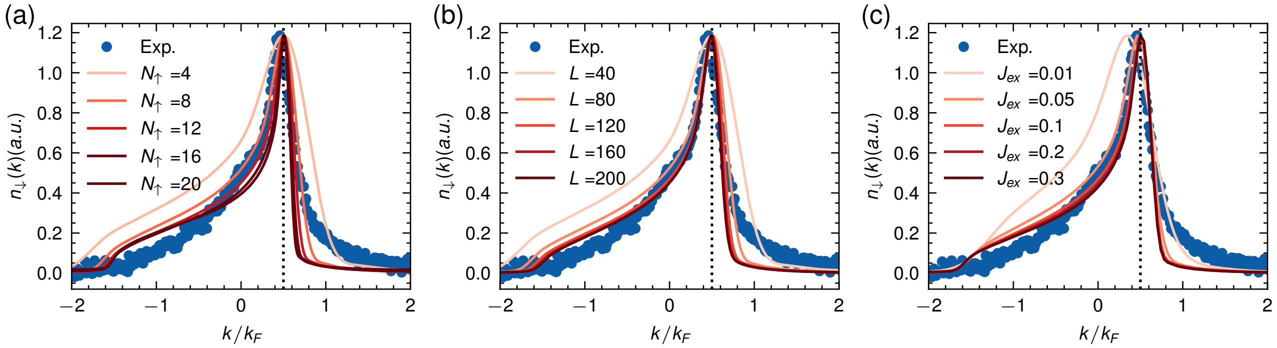

Figure S2. **Effect of different system parameters of the swap model.** (a) Quasi-momentum distribution for the impurity for varying number of host particles $N_\uparrow$ with $J_{\rm ex} = 0.1, L = 40$; (b) Quasi-momentum distribution of impurity for varying system size L with $J_{\rm ex} = 0.1, N_\uparrow/L = 0.1$; (c) Quasi-momentum distribution of impurity for varying $J_{\rm ex}$ with $N_\uparrow = 12, L = 120$. The black dotted lines in (a-c) show the location of the peak, which is given by $k_{\rm peak}/k_{\rm F} = \theta/\pi$. Note that the peak has been rescaled according to the experimental data.

S3. GROUND-STATE PROPERTIES OF THE SWAP MODEL $\hat{H}_{\rm Swap}$

Next, we investigate the effect of various system parameters of the swap model, specifically the number of host particles $N_\uparrow$, the length of the system chain L, and the swapping strength $J_{\rm ex}$. As shown in Fig. S2, changes in these parameters lead to similar behavior, in the sense that larger values give rise to a narrower quasi-momentum distribution (less uncertainty in momentum space). Note that the height of the theoretical quasi-momentum distribution results are rescaled by the experimental data.

Now we fix the system length to $L = 120$, and tune the parameters $J_{\rm ex}$ and $N_\uparrow$. By computing the residuals $\delta = \sum_i |y_i - f(x_i)|$ between experimental data y_i and the model $f(x_i)$, one can see that low values of δ appear in a wide parameter regime, as shown in Fig. S3(b). Similar results can be obtained by using either low filling (e.g. $n = 0.1$) with $J_{\rm ex} = 0.1$ or higher filling (e.g. $n = 0.25$) but lower $J_{\rm ex} = 0.02$, see Fig. S3(a). This trend indicates that in the thermodynamic limit ($N_\uparrow \to \infty$), the optimal value of $J_{\rm ex}$ would tend to 0. The behavior of anyionization in a finite system with open boundary conditions is well captured by using a finite $J_{\rm ex}$ in our swap model.

S4. DYNAMICAL EVOLUTION GOVERNED BY $\hat{H}_{\rm sBHM}$

As mentioned in the main text, a strong host-impurity interaction and a weak force are necessary to achieve anyonization in our system. In the following, we present simulations for which we relax these requirements. We simulate the dynamical evolution by solving the time-dependent Schrödinger equation associated with the spinful Bose-Hubbard Hamiltonian (sBHM). For this simulation, the initial state of the impurity is set to the ground state

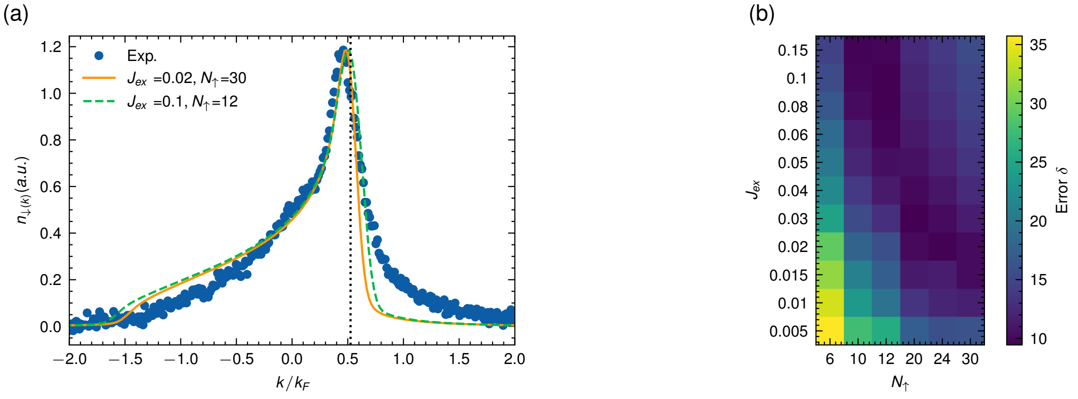

Figure S3. **Comparison between experiment and the predictions of the swap model for different values of $J_{\rm ex}$ and $N_\uparrow$.** (a) Comparison between experimental data (blue dots) and the quasi-momentum distribution obtained by using the swap model for optimal values of the parameters. (b) Residuals between the data and the model as a function of $J_{\rm ex}$ and $N_\uparrow$.

of $\hat{H}_{\rm sBHM} + \frac{V}{2}\sum_\ell \ell^2 \hat{n}_{\downarrow\ell}$, where V quantifies the strength of the impurity harmonic trapping. For weak interactions, the impurity hardly feels the presence of the host particles and drifts away, see the spatio-temporal distribution of the impurity shown in Fig. S4(a). As a result, the quasi-momentum of the impurity is simply shifted from the initial state, in Fig. S4(d) to the final state in Fig. S4(e). In contrast, in the strongly-interacting regime, the impurity hardly moves, i.e., the density is not affected, see Fig. S4(b). The asymmetry in the momentum distribution survives in a wide range of values for the impurity-bath interaction U, which suggests the robustness of the protocol. As the spin wave is a low energy excitation, the force should remain weak to properly prepare it. Indeed, as shown in Fig. S4(c), applying a strong force will result in Bloch oscillations of the impurity[9]. In Fig. S4(f) we see that the force $F_\downarrow a/J$ needs to be kept significantly smaller than 1 to properly reproduce the momentum distribution data.

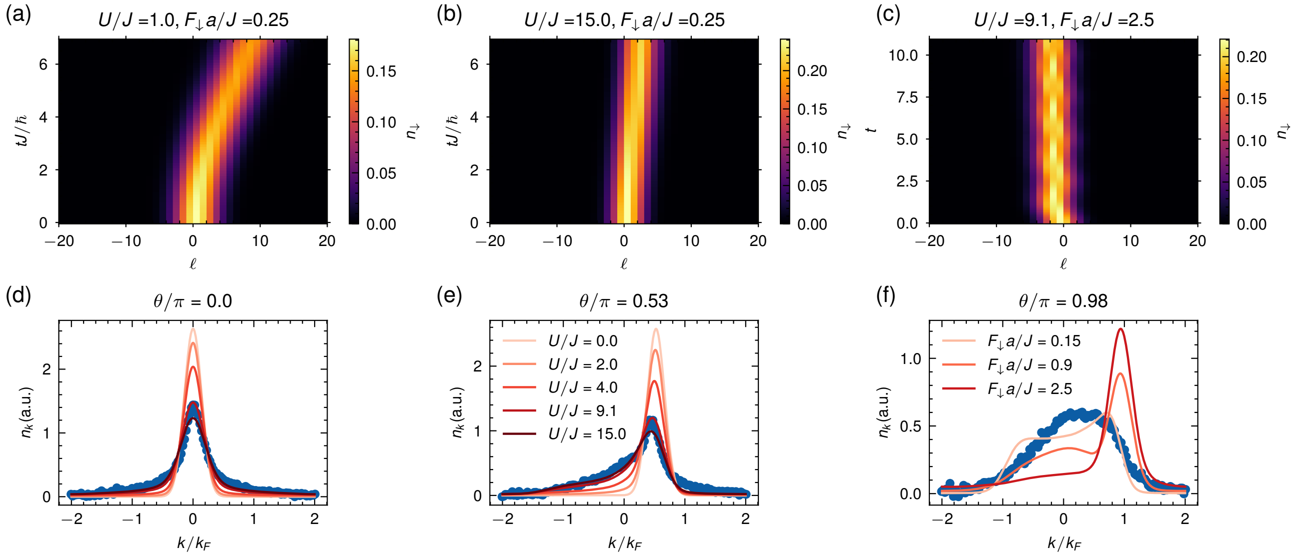

Figure S4. **Effect of the impurity-host interaction U and applied force $F_\downarrow$ as calculated from the sBHM.** (a-c) Spatio-temporal density distribution of the impurity as a function of time for (a) $U/J = 1, F_\downarrow a/J = 0.25$, (b) $U/J = 15, F_\downarrow a/J = 0.25$ and (c) $U/J = 9.1, F_\downarrow a/J = 2.5$. Effect of the impurity-bath interaction in the quasi-momentum distribution of the impurity at (d) $\theta = 0$, (e) $\theta = 0.53\pi$ The same legend applies for (d) and (e). (f) Effect of the force on the quasi-momentum distribution of the impurity at $\theta = 0.98\pi$. Blue dots are experimental data from Fig.2 in the main text and the solid lines are the numerical results. Other parameters are $N_\uparrow = 20, L = 40$ and the initial state is chosen as the ground state of the sBHM with harmonic trap $V/J = 0.02$ applied to the impurity.

We next investigate the effect of the impurity's initial density distribution by subjecting it to a hard-wall confinement of width W (in unit of lattice constant a). Namely, we start with the ground state of the sBHM with an initial hard-

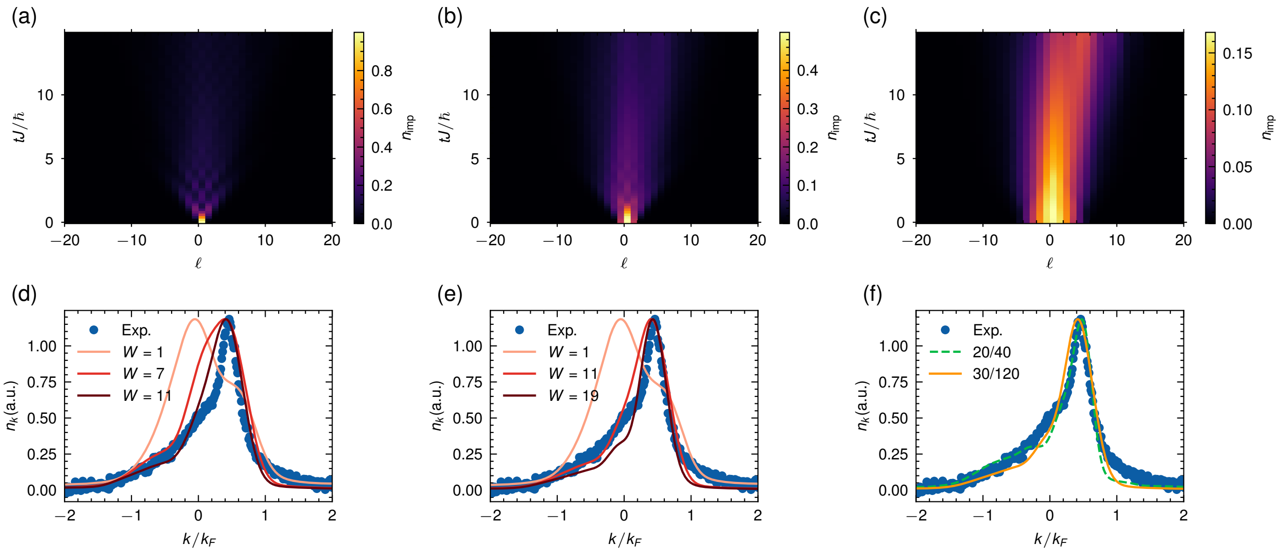

Figure S5. **Effect of the initial density distribution of the impurity determined by a hard-wall confinement.** (a-c) Spatio-temporal density distribution of the impurity for various values of the hard-wall width (a) $W=1$, (b) $W=3$ and (c) $W=11$ in the case of $N_\uparrow=10$ and $L=40$. (d) Comparison of quasi-momentum distribution between the experimental data (blue dots) and the results obtained by using the sBHM (solid lines) for various values for W. (e) Same as (d), but using $N_\uparrow=30$ and $L=120$. (f) Comparison of the quasi-momentum distribution between experimental data (blue dots) and the results obtained from the dynamical evolution governed by the sBHM. The analysis suggests that a decrease in system length can be compensated by considering a larger number of host particles. The other parameters are set to $U/J=4.55$ and $F_\downarrow a/J=0.03125$. Note that in (d-f) the peak has been rescaled according to the experimental data.

wall potential felt only by the impurity and using different values for W. The confinement is then removed for $t\geq 0$. For $W=1$, which corresponds to the impurity initially localized at a single site, i.e., a single Wannier state, the spatial density distribution of the impurity shown in Fig. S5(a) exhibits a decaying breathing pattern. In this case, it is difficult to observe a consistent anyonic feature. For large values of W, the impurity has a broad spatial distribution (see Fig. S5(c)), which thus allows for a proper generation of a spin wave, and the quasi-momentum distribution gets closer to the experimental data; see Fig. S5(d,e). Note that for a long chain ($L=120$) and wide enough initial hard-wall potentials, the time-evolved quasi-momentum distribution agrees with the experimental data, see Fig. S5(e). For a shorter chain ($L=40$), as shown in Fig. S5(e), using a higher host particle density gives rise to similar results.

[1] C. J. M. Mathy, M. B. Zvonarev, and E. Demler, Quantum flutter of supersonic particles in one-dimensional quantum liquids, Nature Physics **8**, 881 (2012).
[2] J. B. McGuire, Interacting fermions in one dimension. i. repulsive potential, Journal of Mathematical Physics **6**, 432 (1965).
[3] T. D. Lee, F. E. Low, and D. Pines, The motion of slow electrons in a polar crystal, Phys. Rev. **90**, 297 (1953).
[4] O. Gamayun, A. G. Pronko, and M. B. Zvonarev, Time and temperature-dependent correlation function of an impurity in one-dimensional Fermi and Tonks–Girardeau gases as a Fredholm determinant, New Journal of Physics **18**, 045005 (2016).
[5] O. Gamayun, O. Lychkovskiy, and M. B. Zvonarev, Zero temperature momentum distribution of an impurity in a polaron state of one-dimensional Fermi and Tonks-Girardeau gases, SciPost Phys. **8**, 053 (2020).
[6] T. Keilmann, S. Lanzmich, I. McCulloch, and M. Roncaglia, Statistically induced phase transitions and anyons in 1D optical lattices, Nature Communications **2**, 361 (2011).
[7] G. Tang, S. Eggert, and A. Pelster, Ground-state properties of anyons in a one-dimensional lattice, New Journal of Physics **17**, 123016 (2015).
[8] G. Tang, S. Eggert, and A. Pelster, Ground-state properties of anyons in a one-dimensional lattice, New Journal of Physics **17**, 123016 (2015).
[9] F. Meinert, M. Knap, E. Kirilov, K. Jag-Lauber, M. B. Zvonarev, E. Demler, and H.-C. Nägerl, Bloch oscillations in the absence of a lattice, Science **356**, 945 (2017).
